# Supplementary material for: Endosymbiotic Fungal Diversity and Dynamics of the Brown Planthopper across Developmental Stages, Tissues, and Sexes Revealed Using Circular Consensus Sequencing
Source: Insects. 2024 Jan 29;15(2):87. doi: 10.3390/insects15020087 (PMC10889434; doi:10.3390/insects15020087)
Supplement: Supplementary file 1 [file insects-15-00087-s001.zip › insects-2713573-supplementary.pdf]

Table S1. Classification of OTUs

Sample	Phylum	Class	Order	Family	Genus	Species
D0	3	16	23	46	79	136
D1	4	15	22	35	50	101
D2	3	16	26	43	60	102
D3	3	14	23	40	55	92
D4	3	17	28	48	70	114
D5	4	14	21	33	47	78
DF	3	11	15	26	38	77
DM	3	12	22	38	57	97
F-Sal	5	18	30	53	109	217
F-Mid	4	14	23	42	67	117
F-Mal	4	16	25	38	57	81
F-Hin	3	15	21	33	46	66
F-Ova	4	13	25	35	52	81
F-Fat	4	16	25	32	51	88
F-Hon	3	15	20	31	47	86
M-Sal	4	13	22	33	46	82
M-Mid	5	17	26	46	67	123
M-Mal	4	15	26	46	70	136
M-Hin	5	16	31	45	65	115
M-Tes	4	13	22	33	49	81
M-Fat	4	14	24	39	58	102
All	5	31	73	146	336	760

D0, D1, D2, D3, D4, D5, DF and DM refer to eggs, 1-5 instar nymphs, female and male adults, respectively. F-Sal F-Mid, F-Mal, F-Hin, F-Ova, F-Fat and F-Hon refer to salivary gland, midgut, malpighian tubule, hindgut, ovary, fat body and honeydew of adult females, respectively. M-Sal, M-Mid, M-Mal, M-Hin, M-Tes and M-Fat refer to salivary gland, midgut, malpighian tubule, hindgut, teste and fat body of adult males, respectively.

Table S2. Relative abundance of dominant fungi of BPH at various developmental stages of BPH at phylum and genus levels.

Taxonomy	group	D0	D1	D2	D3	D4	D5	DF	DM	All
Phylum	Ascomycota	80.36%±5.77%	91.05%±4.89%	97.28%±1.54%	97.49%±1.25%	97.77%±0.61%	98.59%±0.20%	99.47%±0.31%	79.13%±3.60%	92.64%±8.39%
	Basidiomycota	18.97%±6.25%	6.88%±3.55%	2.62%±1.64%	2.30%±0.91%	2.01%±0.43%	1.39%±0.22%	0.48%±0.28%	20.84%±3.58%	6.94%±8.24%
	Chytridiomycota	0.00%±0.00%	0.47%±1.05%	0.00%±0.00%	0.00%±0.00%	0.00%±0.00%	0.00%±0.00%	0.00%±0.00%	0.00%±0.00%	0.06%±0.37%
	Mucoromycota	0.67%±0.63%	1.60%±1.96%	0.10%±0.21%	0.21%±0.46%	0.22%±0.47%	0.02%±0.02%	0.05%±0.08%	0.02%±0.05%	0.36%±0.87%
Genus	Acremonium	2.25%±4.57%	0.27%±0.23%	0.93%±0.60%	1.37%±0.43%	1.89%±0.76%	1.86%±0.46%	0.33%±0.15%	23.97%±7.97%	4.11%±8.19%
	Fusarium	3.08%±3.51%	1.69%±2.16%	0.76%±0.77%	0.37%±0.72%	0.22%±0.30%	0.06%±0.08%	0.02%±0.02%	0.09%±0.08%	0.79%±1.71%
	Hirsutella	8.97%±1.92%	10.54%±0.62%	12.04%±0.81%	11.89%±0.69%	13.67%±0.98%	12.53%±0.29%	13.73%±0.97%	7.17%±1.63%	11.32%±2.40%
	Malassezia	15.23%±7.67%	2.23%±0.81%	1.26%±1.18%	0.81%±0.24%	0.34%±0.35%	0.10%±0.05%	0.14%±0.21%	0.18%±0.16%	2.54%±5.51%
	Metarhizium	0.80%±1.78%	0.06%±0.09%	3.54%±2.29%	4.68%±3.22%	0.10%±0.05%	0.01%±0.02%	1.63%±1.58%	0.19%±0.15%	1.38%±2.26%
	Moesziomyces	0.20%±0.24%	0.42%±0.93%	0.62%±0.62%	0.13%±0.26%	1.09%±0.66%	0.93%±0.25%	0.14%±0.22%	17.91%±3.19%	2.68%±5.94%
	Ophiocordyceps	8.60%±4.46%	9.83%±2.52%	4.50%±1.14%	4.89%±2.63%	0.69%±1.09%	0.21%±0.14%	0.14%±0.06%	0.38%±0.17%	3.65%±4.19%
	Polycephalomyces	46.84%±7.30%	65.57%±6.50%	70.38%±5.37%	71.51%±4.00%	79.83%±2.41%	83.35%±0.73%	83.05%±1.50%	39.4%±6.23%	67.49%±16.17%
	Sarocladium	0.02%±0.04%	0.17%±0.18%	0.76%±1.36%	0.15%±0.22%	0.30%±0.13%	0.17%±0.09%	0.04%±0.04%	3.96%±2.16%	0.70%±1.51%
	Wallemia	0.18%±0.36%	3.46%±2.18%	0.29%±0.28%	0.96%±0.55%	0.39%±0.10%	0.33%±0.09%	0.15%±0.10%	2.52%±0.95%	1.04%±1.43%

Mean ± SE. D0, D1, D2, D3, D4, D5, DF and DM refer to eggs, 1-5 instar nymphs, female and male adults, respectively.

Table S3. Relative abundance of dominant fungi of BPH at various developmental stages of BPH at species level.

Group	D0	D1	D2	D3	D4	D5	DF	DM	All
Acremonium furcatum	0.14%±0.30%	0.06%±0.14%	0.50%±0.45%	0.88%±0.26%	0.98%±0.40%	1.29%±0.60%	0.05%±0.05%	15.9%±3.72%	2.47%±5.30%
Acremonium sp. EN13	1.03%±2.46%	0.07%±0.15%	0.30%±0.37%	0.31%±0.25%	0.84%±0.45%	0.53%±0.15%	0.18%±0.11%	7.34%±4.00%	1.36%±2.78%
Hirsutella proturicola	8.86%±1.91%	8.50%±4.77%	11.95%±0.80%	11.81%±0.68%	13.55%±0.93%	12.41%±0.28%	13.52%±1.00%	7.11%±1.63%	10.96%±2.95%
Malassezia arunalokei	5.76%±3.69%	0.01%±0.02%	0.27%±0.34%	0.05%±0.09%	0.02%±0.02%	0.01%±0.01%	0.09%±0.17%	0.04%±0.04%	0.78%±2.25%
Malassezia restricta	5.68%±3.75%	1.45%±1.02%	0.57%±0.31%	0.51%±0.27%	0.25%±0.24%	0.06%±0.05%	0.06%±0.05%	0.07%±0.10%	1.08%±2.21%
Metarhizium minus	0.80%±1.78%	0.02%±0.03%	3.54%±2.29%	4.68%±3.22%	0.10%±0.05%	0.01%±0.02%	1.63%±1.58%	0.19%±0.15%	1.37%±2.26%
Moesziomyces antarcticus	0.20%±0.24%	0.42%±0.93%	0.62%±0.62%	0.13%±0.26%	1.07%±0.67%	0.92%±0.24%	0.14%±0.22%	17.53%±2.97%	2.63%±5.81%
Ophiocordyceps heteropoda	8.57%±4.48%	8.05%±5.13%	4.49%±1.14%	4.89%±2.63%	0.67%±1.10%	0.20%±0.15%	0.10%±0.06%	0.38%±0.17%	3.42%±4.15%
Polycephalomyces prolificus	46.84%±7.30%	54.34%±30.62%	70.37%±5.37%	71.51%±4.00%	79.83%±2.41%	83.35%±0.73%	83.05%±1.50%	39.40%±6.23%	66.09%±19.31%
Wallemia mellicola	0.16%±0.37%	1.82%±1.76%	0.18%±0.11%	0.90%±0.55%	0.34%±0.09%	0.31%±0.09%	0.13%±0.08%	2.25%±0.94%	0.76%±1.04%

Mean ± SE. D0, D1, D2, D3, D4, D5, DF and DM refer to eggs, 1-5 instar nymphs, female and male adults, respectively.

Table S4. Relative abundance of dominant fungi of female adult BPH tissues at phylum and genus levels.

Taxonomy	group	F-Sal	F-Mid	F-Mal	F-Hin	F-Ova	F-Fat	F-Hon	All
Phylum	Ascomycota	87.17%±8.46%	86.71%±4.13%	86.06%±11.54%	96.81%±1.45%	96.93%±1.90%	99.26%±0.53%	66.25%±29.34%	88.51%±15.48%
	Basidiomycota	3.44%±2.93%	9.18%±3.35%	8.25%±6.98%	2.96%±1.07%	2.95%±1.84%	0.59%±0.37%	33.7%±29.27%	8.71%±14.98%
	Chytridiomycota	0.20%±0.15%	0.00%±0.01%	1.56%±1.87%	0.00%±0.00%	0.05%±0.12%	0.00%±0.01%	0.00%±0.00%	0.27%±0.84%
	Mucoromycota	8.90%±5.88%	4.11%±2.00%	4.13%±7.95%	0.23%±0.43%	0.07%±0.07%	0.14%±0.19%	0.05%±0.10%	2.47%±4.68%
	Zoopagomycota	0.30%±0.66%	0.00%±0.00%	0.00%±0.00%	0.00%±0.00%	0.00%±0.00%	0.00%±0.00%	0.00%±0.00%	0.04%±0.25%
Genus	Acremonium	0.65%±1.32%	0.00%±0.00%	0.01%±0.01%	0.00%±0.00%	0.09%±0.11%	0.04%±0.08%	16.7%±11.49%	2.57%±7.19%
	Cladosporium	0.83%±1.02%	1.27%±1.36%	0.84%±1.19%	0.09%±0.12%	0.21%±0.18%	0.03%±0.04%	5.64%±3.41%	1.27%±2.33%
	Fusarium	3.67%±1.66%	1.19%±0.61%	2.29%±1.94%	0.29%±0.37%	0.21%±0.11%	0.11%±0.08%	17.55%±21.37%	3.69%±9.59%
	Hirsutella	1.41%±0.69%	1.56%±1.30%	0.93%±0.61%	0.56%±0.34%	2.39%±1.47%	10.09%±1.00%	0.02%±0.03%	2.45%±3.40%
	Malassezia	2.61%±2.64%	8.32%±3.48%	6.50%±4.59%	2.71%±1.09%	2.40%±1.12%	0.44%±0.38%	0.10%±0.13%	3.15%±3.53%
	Moesziomyces	0.00%±0.00%	0.00%±0.01%	0.00%±0.00%	0.05%±0.11%	0.00%±0.00%	0.02%±0.02%	26.81%±25.13%	3.95%±13.01%
	Mortierella	8.41%±5.09%	2.41%±3.14%	3.92%±7.53%	0.20%±0.42%	0.05%±0.08%	0.14%±0.19%	0.04%±0.10%	2.16%±4.46%
	Ophiocordyceps	49.45%±14.85%	55.88%±12.88%	63.34%±25.73%	85.43%±2.60%	73.77%±10.61%	19.79%±6.17%	2.96%±2.43%	49.92%±30.37%
	Polycephalomyces	8.68%±5.65%	9.72%±8.72%	6.55%±5.37%	2.99%±1.13%	13.85%±8.20%	66.57%±6.36%	0.03%±0.03%	15.65%±22.50%
	Purpureocillium	6.26%±1.94%	5.35%±1.21%	3.28%±1.38%	5.52%±0.86%	4.62%±0.50%	2.17%±1.21%	0.18%±0.16%	3.87%±2.30%

Mean ± SE. F-Sal, F-Mid, F-Mal, F-Hin, F-Ova, F-Fat and F-Hon refer to salivary gland, midgut, malpighian tubule, hindgut, ovary, fat body and honeydew of adult females, respectively.

Table S5. Relative abundance of dominant fungi of female adult BPH tissues at species level.

Group	F-Sal	F-Mid	F-Mal	F-Hin	F-Ova	F-Fat	F-Hon	All
Acremonium sp. EN13	0.00%±0.00%	0.00%±0.00%	0.00%±0.01%	0.00%±0.00%	0.01%±0.02%	0.01%±0.02%	13.44%±10.54%	1.98%±5.97%
Fusarium equiseti	0.60%±1.33%	0.01%±0.01%	0.00%±0.00%	0.00%±0.00%	0.02%±0.04%	0.01%±0.01%	7.88%±14.11%	1.25%±5.59%
Fusarium sp.	2.38%±1.76%	0.74%±0.42%	2.26%±1.95%	0.00%±0.01%	0.18%±0.14%	0.06%±0.07%	8.82%±16.08%	2.10%±6.32%
Hirsutella proturicola	1.32%±0.69%	1.50%±1.22%	0.92%±0.60%	0.56%±0.34%	2.35%±1.42%	9.90%±0.96%	0.02%±0.03%	2.39%±3.28%
Malassezia restricta	1.32%±1.39%	6.27%±3.55%	3.22%±4.23%	1.12%±0.43%	1.82%±0.78%	0.22%±0.24%	0.07%±0.12%	1.88%±2.66%
Moesziomyces antarcticus	0.00%±0.00%	0.00%±0.01%	0.00%±0.00%	0.05%±0.11%	0.00%±0.00%	0.02%±0.02%	26.62%±24.94%	3.93%±12.73%
Ophiocordyceps heteropoda	49.4%±14.85%	55.86%±12.9%	63.34%±25.73%	85.43%±2.60%	73.76%±10.62%	19.78%±6.15%	2.96%±2.43%	49.9%±29.92%
Polycephalomyces prolificus	8.67%±5.65%	9.71%±8.73%	6.55%±5.37%	2.99%±1.13%	13.85%±8.20%	66.57%±6.36%	0.03%±0.03%	15.65%±22.17%
Purpureocillium lilacinum	5.59%±1.70%	4.74%±1.05%	2.93%±1.23%	4.88%±0.65%	4.14%±0.49%	2.04%±1.14%	0.18%±0.16%	3.46%±2.00%
Pyxidiophora arvernensis	0.00%±0.00%	0.00%±0.00%	0.00%±0.01%	0.00%±0.00%	0.00%±0.00%	0.00%±0.01%	7.22%±16.13%	1.06%±6.09%

Mean ± SE. F-Sal, F-Mid, F-Mal, F-Hin, F-Ova, F-Fat and F-Hon refer to salivary gland, midgut, malpighian tubule, hindgut, ovary, fat body and honeydew of adult females, respectively.

Table S6. Relative abundance of dominant fungi of male adult BPH tissues at phylum and genus levels.

Taxonomy	group	M-Sal	M-Mid	M-Mal	M-Hin	M-Tes	F-Fat	All
Phylum	Ascomycota	73.69%±12.25%	74.20%±2.26%	69.61%±9.17%	77.72%±8.21%	74.14%±11.43%	91.48%±4.46%	76.81%±10.58%
	Basidiomycota	24.09%±10.67%	23.43%±4.89%	26.34%±9.09%	22.08%±8.40%	25.01%±10.95%	8.01%±4.68%	21.5%±9.80%
	Chytridiomycota	0.39%±0.88%	0.43%±0.93%	0.66%±1.47%	0.03%±0.07%	0.15%±0.34%	0.16%±0.33%	0.30%±0.82%
	Mucoromycota	1.83%±2.66%	1.90%±2.77%	3.39%±2.03%	0.01%±0.01%	0.69%±0.65%	0.35%±0.60%	1.36%±2.14%
	Zoopagomycota	0.00%±0.00%	0.03%±0.07%	0.00%±0.00%	0.16%±0.27%	0.00%±0.00%	0.00%±0.00%	0.03%±0.12%
Genus	Aspergillus	0.74%±1.27%	1.61%±1.67%	1.61%±2.24%	11.6%±13.1%	0.29%±0.44%	0.19%±0.20%	2.67%±6.36%
	Cladosporium	0.37%±0.49%	0.27%±0.46%	0.54%±1.07%	4.95%±6.86%	0.75%±0.73%	0.32%±0.35%	1.20%±3.08%
	Eurotium	2.75%±4.97%	0.00%±0.01%	0.00%±0.00%	0.66%±1.27%	0.00%±0.00%	0.17%±0.39%	0.60%±2.15%
	Fusarium	5.47%±3.97%	3.73%±3.56%	6.97%±3.77%	2.53%±3.20%	1.43%±1.82%	0.94%±1.07%	3.51%±3.50%
	Hirsutella	0.54%±0.44%	0.41%±0.34%	1.29%±0.85%	1.43%±1.11%	6.71%±3.11%	10.98%±2.17%	3.56%±4.25%
	Malassezia	23.47%±10.71%	21.81%±3.65%	24.39%±8.31%	16.45%±9.83%	23.72%±10.61%	7.05%±5.12%	19.48%±9.80%
	Mortierella	1.83%±2.66%	1.89%±2.76%	3.35%±2.00%	0.00%±0.00%	0.69%±0.66%	0.34%±0.60%	1.35%±2.13%
	Ophiocordyceps	51.04%±10.84%	53.68%±5.11%	42.77%±5.33%	32.11%±13.52%	20.04%±8.80%	7.66%±3.49%	34.55%±18.31%
	Polycephalomyces	3.34%±2.78%	2.05%±1.07%	4.85%±2.65%	9.02%±3.64%	41.41%±19.17%	68.12%±4.98%	21.46%±26.13%
	Purpureocillium	3.42%±0.96%	4.82%±0.88%	6.63%±2.90%	3.42%±3.04%	1.60%±0.63%	0.64%±0.34%	3.42%±2.56%

Mean ± SE. M-Sal, M-Mid, M-Mal, M-Hin, M-Tes and M-Fat refer to salivary gland, midgut, malpighian tubule, hindgut, teste and fat body of adult males, respectively.

Table S7. Relative abundance of dominant fungi of male adult BPH tissues at species level.

Group	M-Sal	M-Mid	M-Mal	M-Hin	M-Tes	M-Fat	All
Aspergillus ruber	0.03%±0.04%	0.00%±0.00%	0.01%±0.01%	6.71%±8.32%	0.00%±0.00%	0.08%±0.16%	1.14%±4.22%
Cladosporium tenuissimum	0.27%±0.46%	0.05%±0.07%	0.02%±0.04%	3.61%±4.29%	0.44%±0.52%	0.14%±0.17%	0.76%±2.19%
Fusarium sp.	3.17%±3.93%	2.59%±2.17%	4.23%±2.10%	1.07%±1.32%	0.52%±0.76%	0.58%±0.64%	2.03%±2.58%
Hirsutella proturicola	0.53%±0.38%	0.41%±0.30%	1.29%±0.76%	1.41%±0.98%	6.62%±2.73%	10.89%±1.91%	3.52%±4.21%
Malassezia globosa	1.77%±1.95%	2.80%±1.85%	3.89%±2.40%	2.18%±1.44%	3.75%±2.03%	2.12%±1.38%	2.75%±2.06%
Malassezia restricta	14.43%±7.93%	14.60%±3.32%	17.09%±4.06%	13.09%±6.64%	16.35%±5.94%	3.81%±1.75%	13.23%±7.02%
Malassezia sympodialis	6.46%±3.86%	4.23%±3.21%	3.19%±2.51%	0.93%±1.32%	3.06%±2.64%	0.98%±1.66%	3.14%±3.59%
Ophiocordyceps heteropoda	51.04%±9.70%	53.68%±4.57%	42.75%±4.78%	32.11%±12.10%	20.03%±7.89%	7.64%±3.15%	34.54%±18.31%
Polycephalomyces prolificus	3.34%±2.49%	2.05%±0.96%	4.85%±2.37%	9.02%±3.25%	41.41%±17.14%	68.12%±4.45%	21.46%±26.13%

Mean ± SE. M-Sal, M-Mid, M-Mal, M-Hin, M-Tes and M-Fat refer to salivary gland, midgut, malpighian tubule, hindgut, teste and fat body of adult males, respectively.

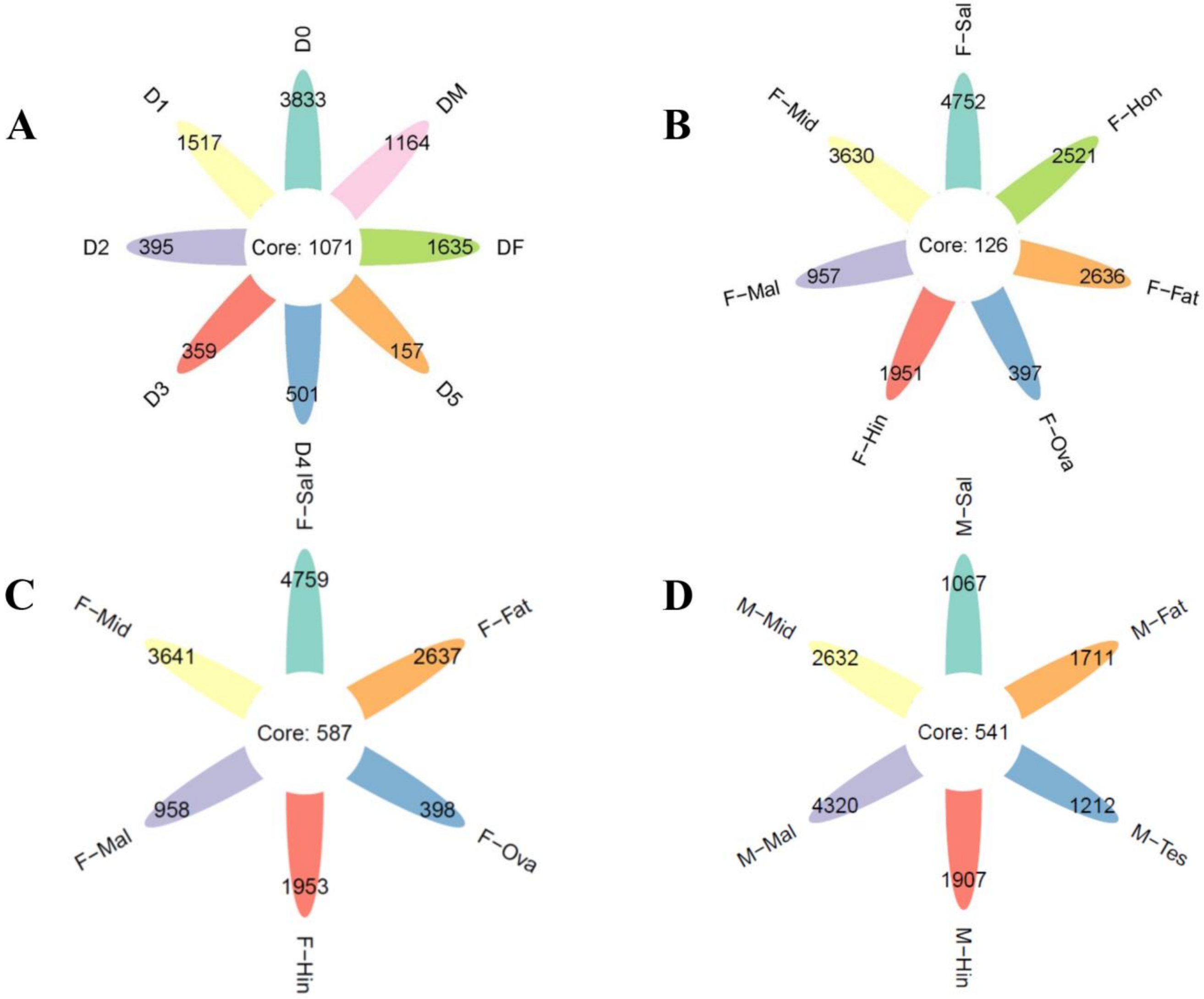

Figure S1. Venn diagram of shared and unique OTUs. D0, D1, D2, D3, D4, D5, DF and DM refer to eggs, 1-5 instar nymphs, female and male adults, respectively. F-Sal, F-Mid, F-Mal, F-Hin, F-Ova, F-Fat and F-Hon refer to salivary gland, midgut, malpighian tubule, hindgut, ovary, fat body and honeydew of adult females, respectively. M-Sal, M-Mid, M-Mal, M-Hin, M-Tes and M-Fat refer to salivary gland, midgut, malpighian tubule, hindgut, teste and fat body of adult males, respectively.

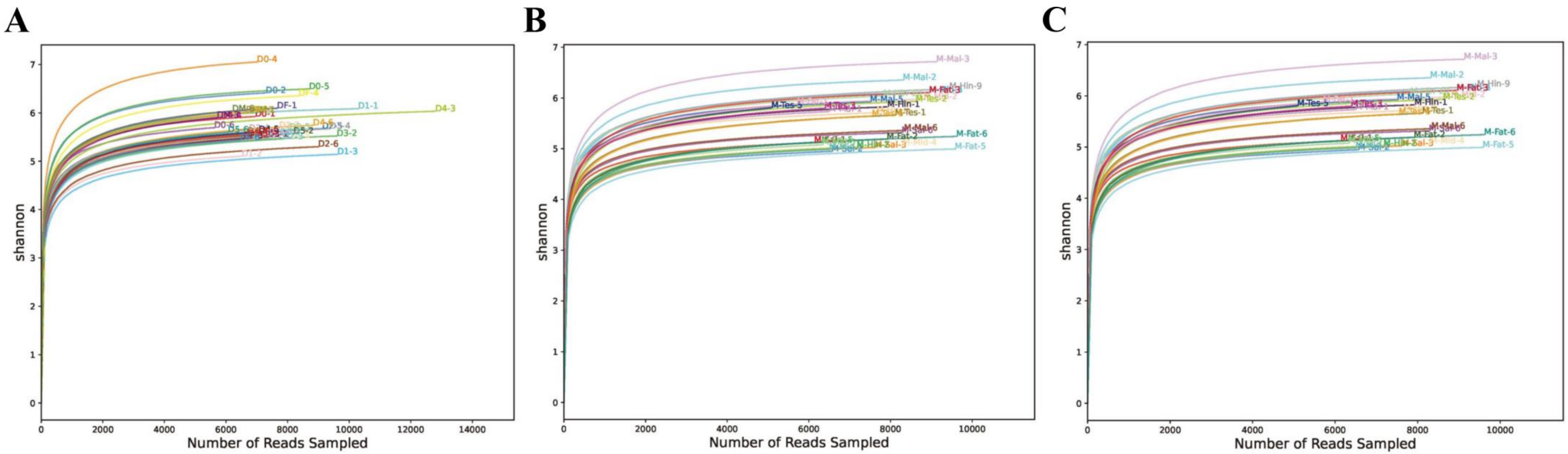

Figure S2. Shannon-winner curves. (A) D0, D1, D2, D3, D4, D5, DF and DM refer to eggs, 1-5 instar nymphs, female and male adults, respectively. (B) F-Sal, F-Mid, F-Mal, F-Hin, F-Ova, F-Fat and F-Hon refer to salivary gland, midgut, malpighian tubule, hindgut, ovary, fat body and honeydew of adult females, respectively. (C) M-Sal, M-Mid, M-Mal, M-Hin, M-Tes and M-Fat refer to salivary gland, midgut, malpighian tubule, hindgut, teste and fat body of adult males, respectively.

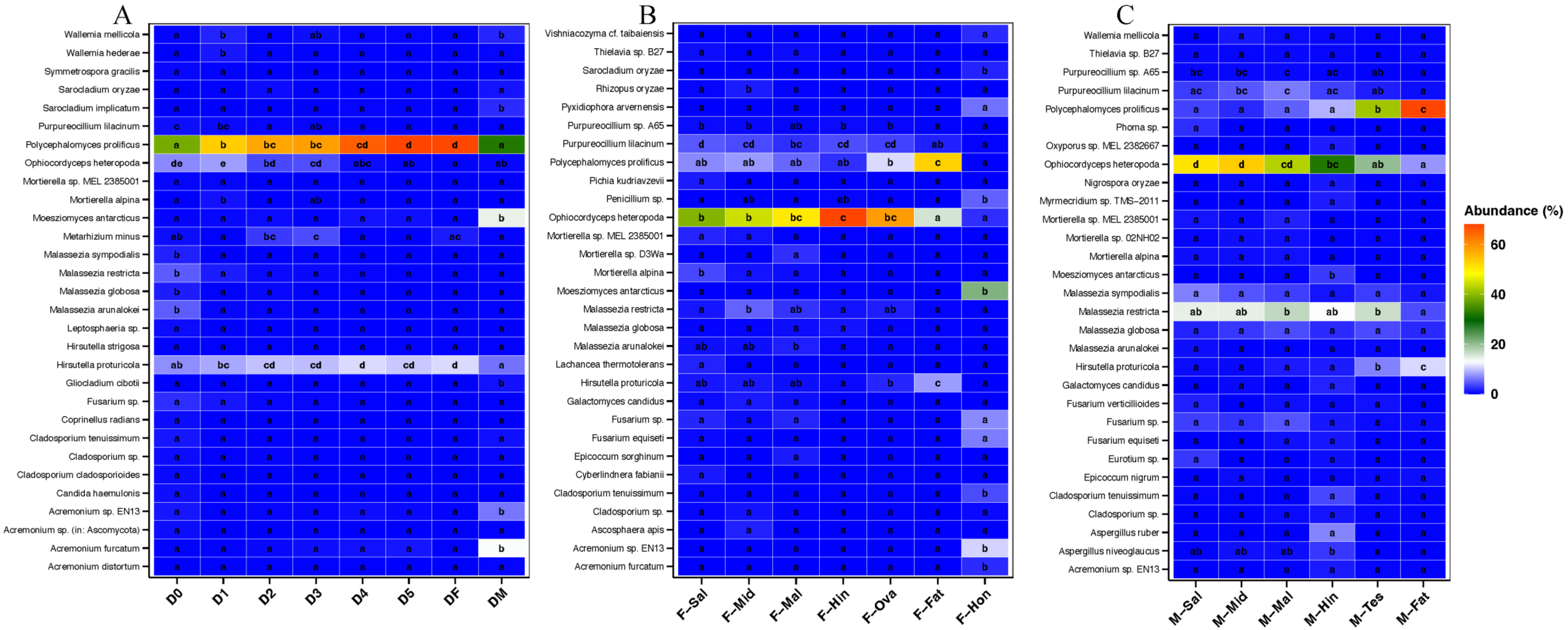

Figure S3. Heatmap of the top 30 species of relative abundance at the species level. Tukey 's HSD test was used to test the difference between groups. The different letters above each group of samples represent significant differences between groups. (A) D0, D1, D2, D3, D4, D5, DF and DM refer to eggs, 1-5 instar nymphs, female and male adults, respectively. (B) F-Sal, F-Mid, F-Mal, F-Hin, F-Ova, F-Fat and F-Hon refer to salivary gland, midgut, malpighian tubule, hindgut, ovary, fat body and honeydew of adult females, respectively. (C) M-Sal, M-Mid, M-Mal, M-Hin, M-Tes and M-Fat refer to salivary gland, midgut, malpighian tubule, hindgut, teste and fat body of adult males, respectively.

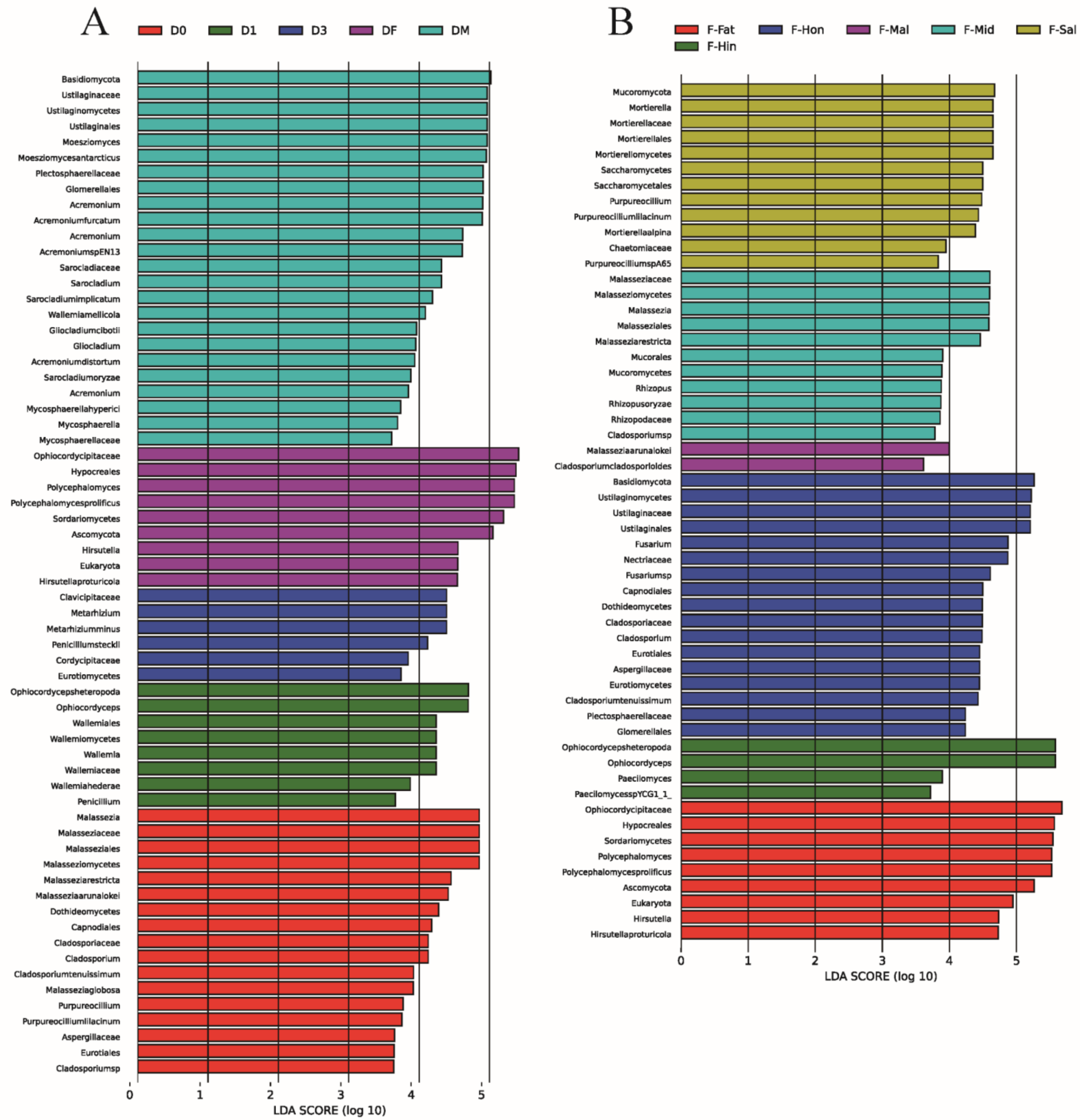

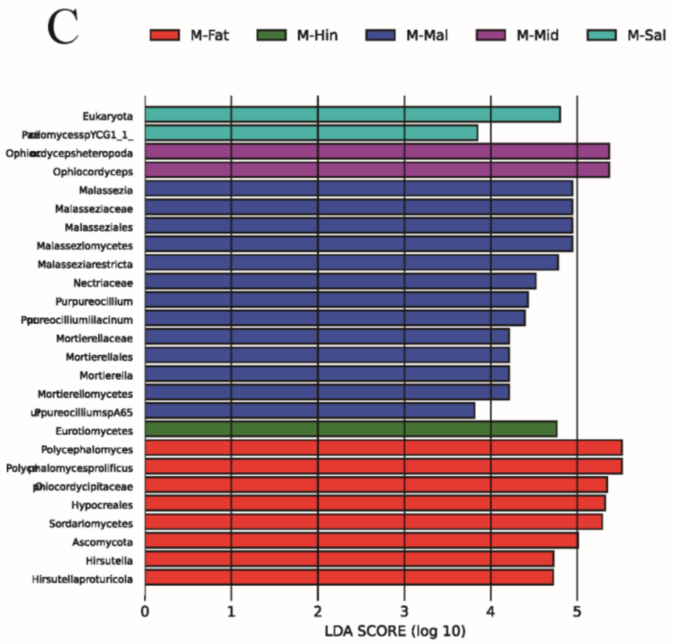

Figure S4. Histogram of LDA values > 3. (A) D0, D1, D2, D3, D4, D5, DF and DM refer to eggs, 1-5 instar nymphs, female and male adults, respectively. (B) F-Sal, F-Mid, F-Mal, F-Hin, F-Ova, F-Fat and F-Hon refer to salivary gland, midgut, malpighian tubule, hindgut, ovary, fat body and honeydew of adult females, respectively. (C) M-Sal, M-Mid, M-Mal, M-Hin, M-Tes and M-Fat refer to salivary gland, midgut, malpighian tubule, hindgut, teste and fat body of adult males, respectively.

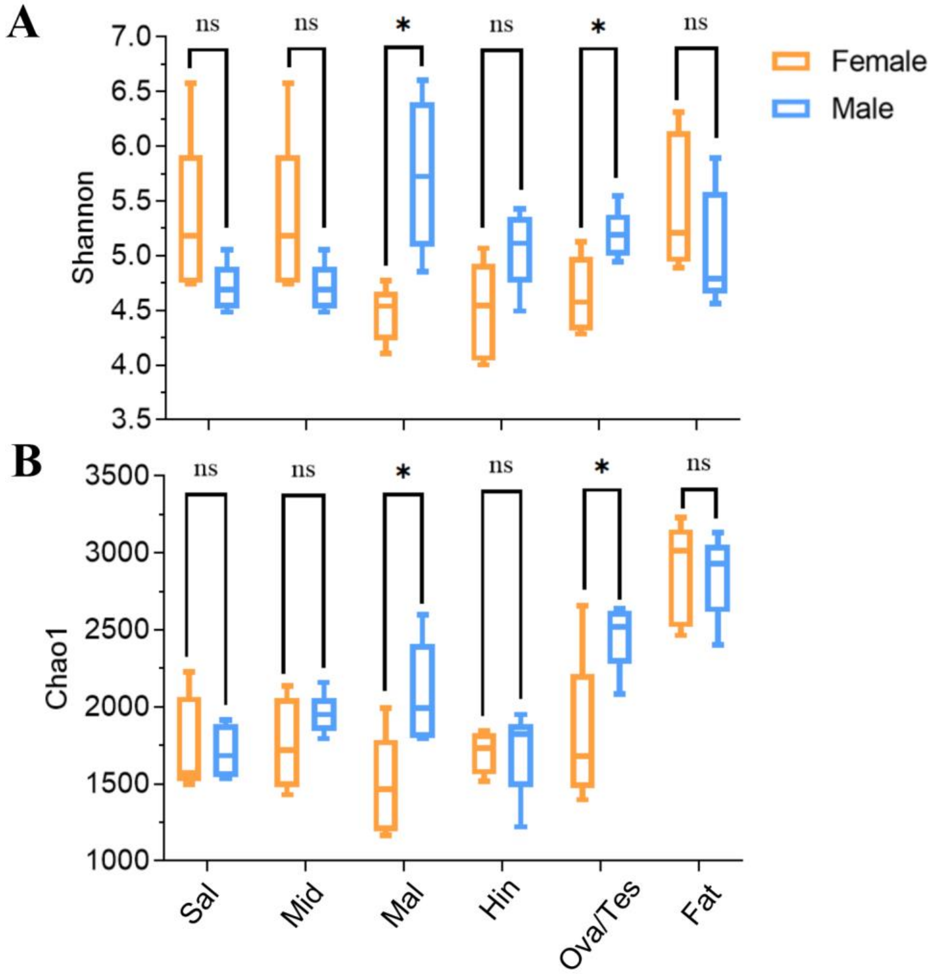

Figure S5. Chao1 (A) and Shannon (B) indices of Alpha analysis in different tissues between females and males. Sal, Mid, Mal, Hin, Ova/Tes and Fat refer to salivary gland, midgut, malpighian tubule, hindgut, ovary/teste and fat body, respectively.

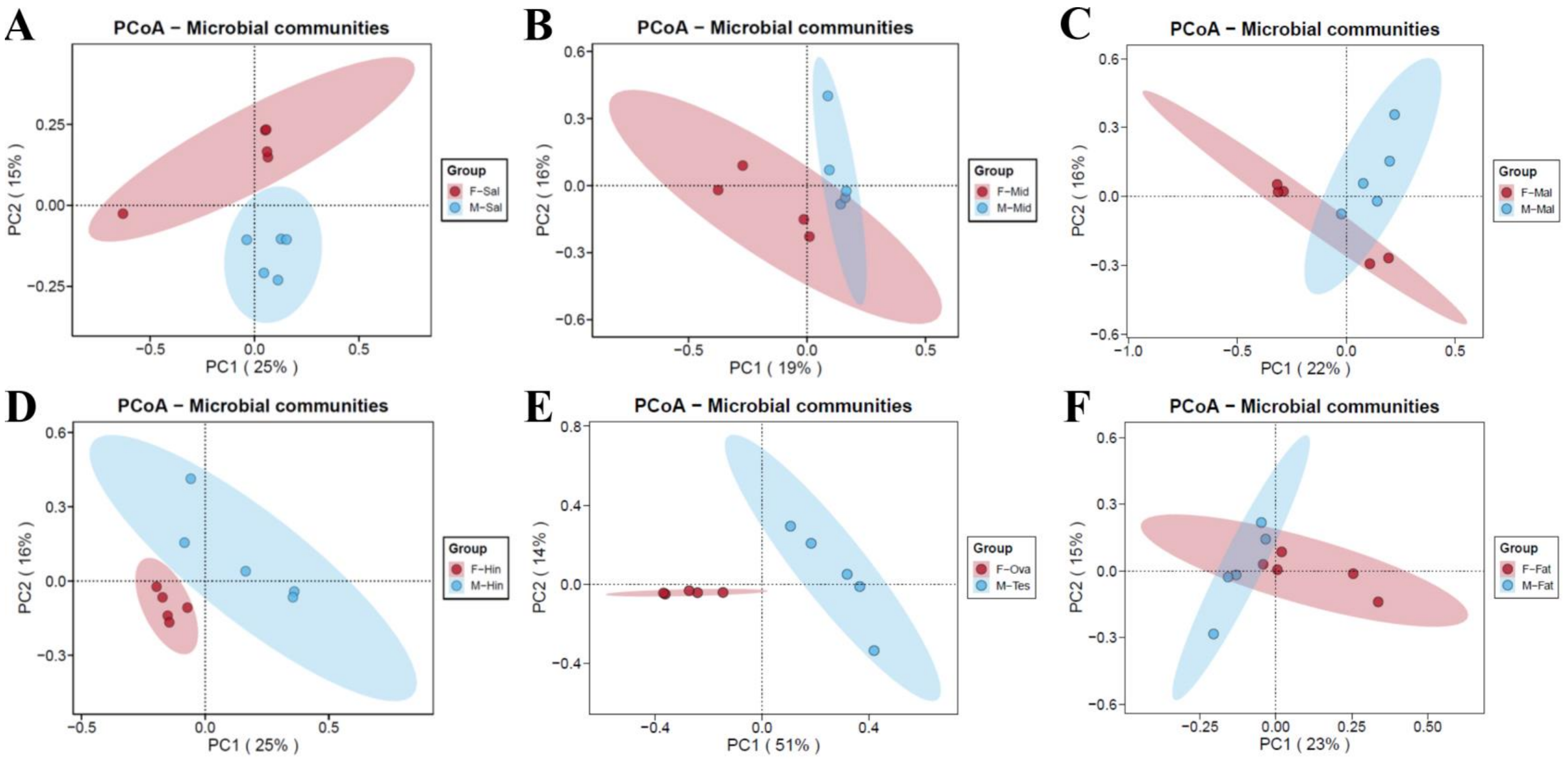

Figure S6. PCoA plots of Bray-Curtis distances for fungal communities of the various tissues between females and males. F-Sal (A), F-Mid (B), F-Mal (C), F-Hin (D), F-Ova (E), F-Fat (F) refer to salivary gland, midgut, malpighian tubule, hindgut, ovary, fat body of adult females, respectively. M-Sal (A), M-Mid (B), M-Mal (C), M-Hin (D), M-Tes (E) and M-Fat (F) refer to salivary gland, midgut, malpighian tubule, hindgut, teste and fat body of adult males, respectively.
